# Comorbid asthma – occurrence and associations with patient-related outcomes in COPD: a cohort study

**DOI:** 10.1080/02813432.2026.2747915

**Published:** 2026-10-18

**Authors:** Åsa Athlin, Karin Lisspers, Mikael Hasselgren, Björn Ställberg, Hanna Sandelowsky, Maaike Giezeman, Christer Janson, Marta A. Kisiel, Gabriella Eliason, Anna Nager, Scott Montgomery, Josefin Sundh

**Affiliations:** a School of Medical Sciences, Faculty of Medicine and Health, Örebro University, Örebro, Sweden; b Department of Public Health and Caring Sciences, General Practice, Uppsala University, Uppsala, Sweden; c Centre for Clinical Research and Education, Karlstad, Sweden; d Department of Neurobiology, Care Sciences and Society, Division of Family Medicine and Primary Care, Karolinska Institute, Stockholm, Sweden; e Academic Primary Care Centre, Stockholm, Sweden; f Department of Medical Sciences, Respiratory, Allergy and Sleep Research, Uppsala University, Uppsala, Sweden; g Department of Medical Sciences, Occupational and Environmental Medicine, Uppsala University, Uppsala, Sweden; h Department of Respiratory Medicine, Faculty of Medicine and Health, Örebro University, Örebro, Sweden; i Clinical Epidemiology and Biostatistics, School of Medical Sciences, Faculty of Medicine and Health, Örebro University, Örebro, Sweden; j Clinical Epidemiology Division, Department of Medicine, Karolinska Institute, Stockholm, Sweden; k Department of Epidemiology and Public Health, University College, London, UK

**Keywords:** Primary care, real-life, mortality, exacerbation, clinical COPD questionnaire, Comorbid asthma

## Abstract

**Purpose:**

Clinical features of COPD and asthma overlap, complicating differential diagnosis. This study investigated the prevalence of comorbid asthma in COPD patients and its association with patient-related outcomes.

**Materials and methods:**

Patients with a doctor’s diagnosis of COPD were randomly selected in two cohorts 2005 (*n* = 1060) and 2014 (*n* = 1671), from primary and secondary care in Sweden. Data were collected from medical records and patient questionnaires at baseline and at follow-up (2012 and 2022, respectively). Questionnaires included data on exacerbations. Symptoms were assessed using the clinical COPD questionnaire (CCQ). Comorbid asthma was identified through medical records. Ten-year mortality data were obtained from national registers. Logistic and linear regression assessed associations with patient-reported outcomes; Cox regression was applied for mortality analyses.

**Results:**

Comorbid asthma was found in 22% of the 2005 cohort and 9% of the 2014 cohort (*p* < .001). In both cohorts, it was associated with an increased risk of exacerbations: OR (95% CI) 1.58 (1.07–2.32), *p* = .02, and 2.20 (1.38–3.52), *p* < .001, respectively. No associations were found between comorbid asthma and worsening of health status (CCQ). Mortality risk did not differ in the 2005 cohort, HR (95% CI) 0.92 (0.71–1.18), but was lower in the 2014 cohort, HR (95% CI) 0.67 (0.49–0.93), *p* = .02.

**Conclusion:**

Comorbid asthma in COPD has changed from 22 to 9%, potentially due to improved diagnostics. Comorbid asthma remains clinically relevant due to its association with an increased exacerbation risk.

## Introduction

Chronic obstructive pulmonary disease (COPD) and asthma are common chronic diseases in the general population. In Sweden, these conditions are most often diagnosed and managed in primary care [1,2]. In a typical patient, clinical characteristics and diagnostic criteria are well-defined [3,4]. However, distinguishing between the two diseases can be challenging, as they may present with similar symptoms and overlapping characteristics. For example, the asthma phenotype with fixed obstruction has a different pathogenesis than COPD and the pathophysiology differs in the lack of emphysema. Conversely, a patient with COPD may exhibit asthma characteristics, such as a bronchodilator response and elevated levels of blood eosinophils [5,6].

The term asthma-COPD overlap (ACO) has been suggested to describe patients who present with features of both conditions [3] but there is currently no consensus on how to define this heterogeneous condition [7,8], which complicates the comparison of findings from different studies [9].

Previous studies on patients with a doctor’s diagnosis of COPD have shown that the prevalence of a concurrent asthma diagnosis varies, ranging from 15 to 27%. This variation depends on difference in methodology, diagnostic criteria and the inclusion of spirometry data [10–14]. Several population-based studies have shown that patients with clinical features of both COPD and asthma are at an increased risk of more frequent and severe exacerbations compared to those with only one of the diseases [15–19]. However, reported data on mortality and health related quality of life have shown conflicting results [11,20–26].

There is a lack of real-world studies from primary care and hospital out-patient clinics that address prevalence and patient-related outcomes in patients with COPD and comorbid asthma. Several studies apply strict inclusion criteria, often excluding participants with comorbid asthma, among other conditions [27,28]. This has raised concerns about the generalizability of the findings to a real-world population, highlighting the need to complement the evidence with data from patients treated in routine care [28].

The aim of this study was to describe the prevalence of comorbid asthma in a population of patients from two COPD cohorts, randomly selected nine years apart in the same geographic area in Sweden, with a population largely from primary care. Furthermore, we sought to explore associations between comorbid asthma and exacerbations, health status and mortality in patients with COPD.

## Material and methods

### Study design and population

The PRAXIS study, initiated in 2005, is a long-term research project aimed at investigating asthma and COPD in a real-world setting. It includes both primary and secondary care in a defined geographical region in central Sweden. The study consists of two cohorts, PRAXIS I and PRAXIS II, established in 2005 and 2014, respectively. Several previously published articles have, among other aspects, studied the associations between obstructive lung disease and various comorbidities, health related quality of life and mortality [29–31].

This study includes the two COPD cohorts of the PRAXIS study.

The Healthcare Region in central Sweden has a total population of ∼2.1 million and consists of seven county councils, each represented in the study.

Patients were enrolled from seven central hospitals, seven randomly selected district hospitals and 56 randomly selected primary health care centers (PHCCs) in the 2005 cohort (PRAXIS I). In the 2014 cohort (PRAXIS II), patients were enrolled from six of the central and six of the district hospitals that had participated in PRAXIS I, together with 75 randomly selected PHCCs.

### Patient selection and data collection

The patient selection and data collection are presented in Figure 1. At each medical unit all listed patients with a doctor’s diagnosis of COPD (ICD-code J44) in the medical records during the periods 2000–2003 (PRAXIS I) and 2007–2010 (PRAXIS II), were retrieved and an internet-based random selection was performed (www.random.org). The selected patients received a questionnaire for self-completion at baseline (2005 and 2014, respectively) and at the follow-ups of each cohort (2012 and 2022, respectively). A letter of consent was collected at study start. Medical records on each patient were collected from the periods 2000–2003 (PRAXIS I) and 2004–2014 (PRAXIS II). Two research nurses performed reviews of the medical records using a specific template. Patients with a concomitant diagnosis of asthma were identified either by a doctor’s diagnosis (ICD-code J45) or if clearly stated in the medical record at any time. No further verification of either COPD or asthma diagnoses were performed. Patients that were identified to have had a change of diagnosis from asthma to COPD were categorized as ‘COPD without asthma’. Mortality data were obtained from the Cause of Death Register at the National Swedish Board of Health and Welfare up till 31 July 2015 (PRAXIS I) and 31 December 2024 (PRAXIS II).

**Figure 1.**
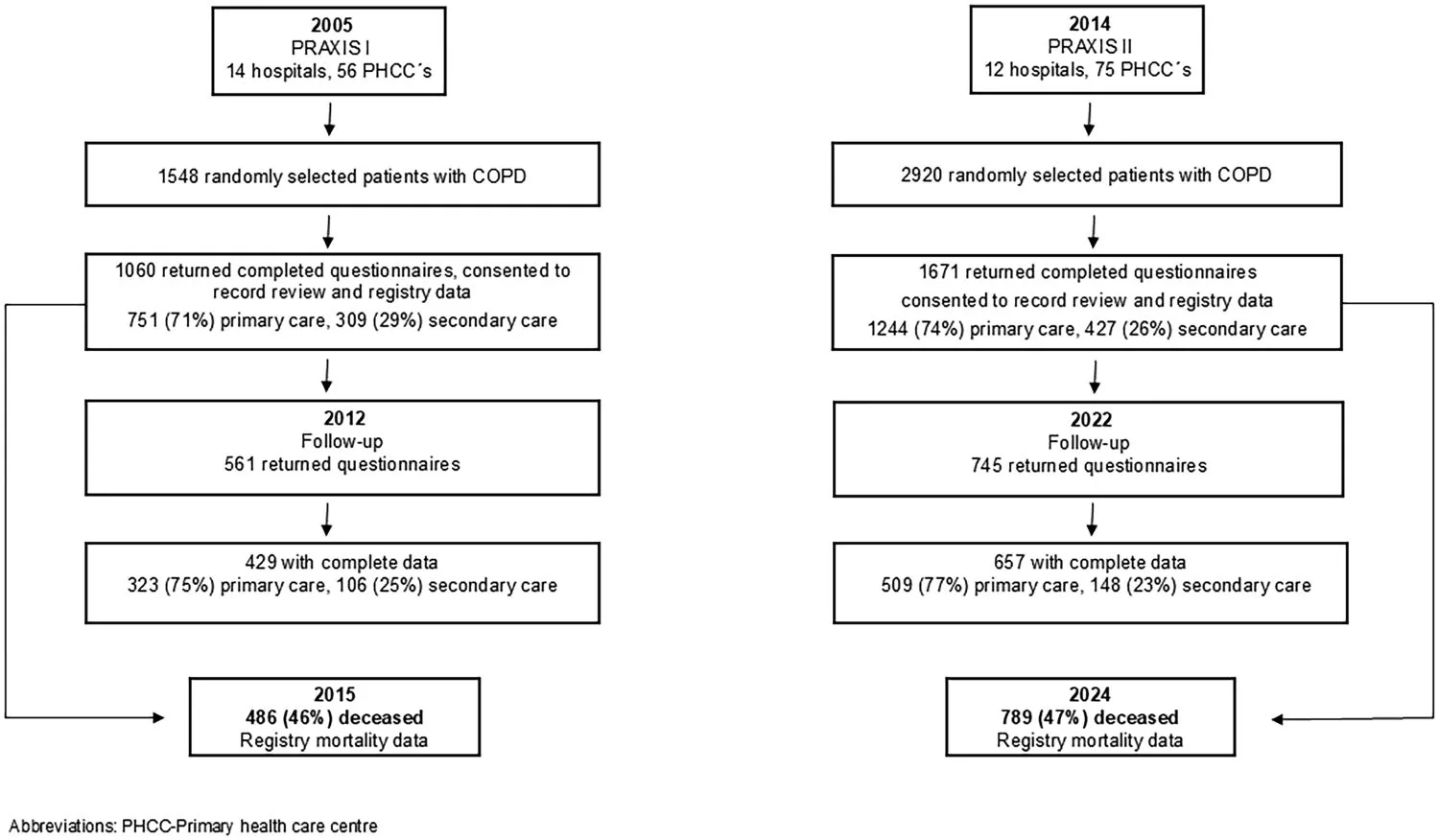
Flow chart of patient selection and data collection. PHCC: primary health care center.

#### Measures and variables in the study

The patient questionnaires were designed specifically for the PRAXIS study and consisted of 43 (PRAXIS I) and 67 items (PRAXIS II), respectively. In this study, we extracted data on patient characteristics and outcomes including:

- Age at baseline categorized as ≤60, 61–65, 66–70 and >70 years.
- Body mass index (BMI) categorized as <20, 20–24.9, 25–29.9 and ≥30 kg/m^2^.
- Smoking status categorized as never smoker, ex-smoker, occasional smoker and current daily smoker.
- Use of inhaled corticosteroids (ICS) regularly.
- Exacerbations, defined as emergency visits or a course of oral corticosteroids with or without antibiotics due to worsening of COPD, during the previous six months.
- Health status assessed by the clinical COPD questionnaire (CCQ). The questionnaire consists of 10 items distributed over three domains based on experiences the previous week: symptoms, mental state and functional state. Each item is scored from 0 to 6. All scores are added, and the sum divided by 10. The total mean CCQ-score varies from 0 (very good control) to 6 (extremely poor control) [24].

Data on lung function and comorbidities were obtained from medical record review for the periods described above and included:

- Lung function measured by spirometry expressed as forced expiratory volume in 1 s in percent of predicted (FEV_1_% pred) categorized in stages according to GOLD as <30%, 30–49%, 50–79% and ≥80% [4].
- Heart disease, defined as a diagnosis of ischemic heart disease or chronic heart failure.
- Diabetes, defined as a diagnosis of types 1 or 2 diabetes mellitus.
- Depression, defined as a diagnosis with current antidepressant drug treatment.

### Statistical analyses

All analyses were performed using IBM SPSS version 29.0 (IBM Corporation, Armonk, NY).

Categorical data were expressed by frequencies and percentages, and continuous data as means and standard deviations or medians and interquartile range (IQR) if not normally distributed. The chi-squared test and students *t*-test were used to analyze the differences in proportions and means, respectively, between patients with COPD with and without a diagnosis of comorbid asthma.

Linear regression was used to assess associations of comorbid asthma with mean CCQ as dependent variable at follow-up in 2012 and 2022, respectively. Results were presented as regression coefficients with 95% confidence intervals (95% CI). To assess the association of comorbid asthma with exacerbations at the follow-ups, logistic regression was used. The results were presented as odds ratios (OR) with 95% CI. Cox regression was used to explore the association with all-cause mortality. Results were presented as hazard ratios (HR) with 95% CI. In all analyses a *p* value <.05 was considered significant. The models were adjusted for age, sex, BMI (four groups), smoking (four groups), exacerbations or not during the previous six months, CCQ mean score and comorbid heart disease at baseline. As sensitivity analyses, the main models were repeated with additional adjustment for treatment with ICS. The potential confounders were chosen based on previous knowledge on factors associated with patient-related outcomes in COPD [29,32,33].

## Results

### Patient characteristics

Patient characteristics at baseline for both cohorts stratified by comorbid asthma are presented in Table 1.

**Table 1.** Patient characteristics at baseline for PRAXIS cohorts I (2005) and II (2014) stratified by COPD with and without comorbid asthma.

	PRAXIS I 2005 cohort *n* = 1060			PRAXIS II 2014 cohort *n* = 1671		
Age (years) median (IQR)	66.0 (60–70)			69.0 (64–72)		
Sex m/f *n* (%)	444 (42)/616 (58)			734 (44)/937 (56)		
	COPD without asthma, *n* (%) 827 (78)	COPD with asthma, *n* (%) 233 (22)	*p* Value	COPD without asthma, *n* (%) 1520 (91)	COPD with asthma, *n* (%) 151 (9)	*p* Value
Age (years) median (IQR)	66.0 (61–70)	65.0 (59–70)		69.0 (64–73)	67.0 (61–71)	–
Age, years	–	–	0.07	–	–	0.018
≤60	193 (23)	74 (32)		221 (15)	34 (22)	
61–65	208 (25)	50 (22)		234 (15)	28 (19)	
66–70	222 (27)	59 (25)		486 (32)	46 (31)	
>70	204 (25)	50 (21)		579(38)	43 (29)	
Sex *n* (%)	–	–	<0.001	–	–	0.28
Male	370 (45)	74 (32)	–	674 (44)	60 (40)	
Female	457 (55)	159 (68)	–	846(56)	91 (60)	
Care level	–	–	0.26	–	–	0.91
Primary care	579 (70)	172 (74)	–	1131 (74)	113 (75)	
Secondary carè	248 (30)	61(26)	–	389 (26)	38 (25)	
Smoking status^a^	–	–	0.06	–	–	0.51
Never smoker	44 (5)	23 (10)		62 (4)	10 (7)	
Ex-smoker	481 (58)	133 (57)		940 (63)	95 (63)	
Occasional smoker	61 (7)	12 (5)		91 (6)	9 (6)	
Current daily smoker	240 (29)	64 (28)		396 (27)	36 (24)	
Heart disease	195 (24)	65 (28)	0.18	342 (24)	29 (19)	0.15
Hypertension	223 (27)	67 (29)	0.59	738 (53)	70 (46)	0.14
Diabetes	75 (9)	26 (11)	0.34	239 (16)	36 (24)	0.01
Depression	99 (12)	28 (12)	0.99	358 (25)	39 (26)	0.95
FEV_1_ % pred^b^	–	–	0.51	–	–	0.41
<30%	48 (11)	8 (8)	–	122 (10)	8 (6)	
30–49%	126 (29)	26 (25)		331 (26)	34 (26)	
50–79%	179 (42)	49 (47)		626 (50)	66 (50)	
≥80%	78 (18)	21 (20)	–	173 (14)	23 (18)	
Body mass index^c^	–	–	0.024	–	–	0.28
<20	99 (12)	15 (7)	–	128 (9)	12 (8)	
20–24.9	280 (35)	83 (37)	–	481 (33)	44 (30)	
25–29.9	277 (35)	72 (32)	–	513 (35)	45 (31)	
≥30	144 (18)	55 (24)	–	346 (24)	45 (34)	
Inhaled corticosteroids regularly	434 (53)	183 (79)	<0.001	814(54)	119 (79)	<0.001
Exacerbations at baseline	–	–	<0.001	–	–	<0.001
Yes	287 (35)	108 (46)	–	467 (31)	67 (44)	–
No	540 (65)	125 (54)	–	1053 (69)	84 (56)	
CCQ at baseline^d^ mean (SD)	2.20 (1.35)	2.36 (1.36)	0.88	2.01 (1.29)	1.97 (1.30)	0.87

Notes: missing data: ^a^*n* = 1058 PRAXIS I, *n* = 1639 PRAXIS II, ^b^*n* = 535 PRAXIS I, *n* = 1383 PRAXIS II, ^c^*n* = 1025 PRAXIS I, *n* = 1614 PRAXS II, ^d^*n* = 923 PRAXIS I, *n* = 1394 PRAXIS II.

IQR: interquartile range; FEV_1_% pred: forced expiratory volume in 1 s in percent of predicted; CCQ: clinical COPD questionnaire; SD: standard deviation.

Definitions: heart disease – chronic heart failure and/or ischemic heart disease, diabetes – types 1 and 2 diabetes, depression – recorded diagnosis with concurrent medication.

In the PRAXIS I cohort, median age was 66 years (IQR 60–70), 58% were women and most patients were from primary care (71%). In the PRAXIS II cohort, the median age was 69 years (IQR 64–72), 56% were women and 74% recruited from primary care.

### Prevalence of comorbid asthma in patients with COPD

The prevalence of patients with COPD and comorbid asthma was 22% in the PRAXIS I cohort and 9% in the PRAXIS II cohort (Table 1). The difference was statistically significant (*p* < .001). In PRAXIS I concomitant asthma was present in 22.9% of patients in primary care and in 19.7% of patients in secondary care. In PRAXIS II the corresponding proportions were 9.1 and 8.9%, respectively. No statistical differences in proportion of comorbid asthma between primary and secondary care were observed in either PRAXIS I (*p* = .295) or PRAXIS II (*p* = .987).

### Patient-related outcomes

At follow-up, COPD with comorbid asthma was independently associated with an increased risk of exacerbations in both cohorts. No associations between COPD with comorbid asthma and worsening of health status (CCQ) in either cohort were found (Table 2 and Figure 2). This finding remained when analyses were performed on each CCQ domain (data not shown). Sensitivity analyses with adjustment for treatment with ICS did not alter the result substantially. After 10 years follow-up, mortality in the PRAXIS I cohort was 46% (*n* = 486) and in the PRAXIS II cohort 47% (*n* = 789). COPD with comorbid asthma was not associated with a higher risk of mortality in the PRAXIS I cohort whereas in the PRAXIS II cohort comorbid asthma was associated with a decreased mortality risk (Table 2 and Figure 2).

**Figure 2.**
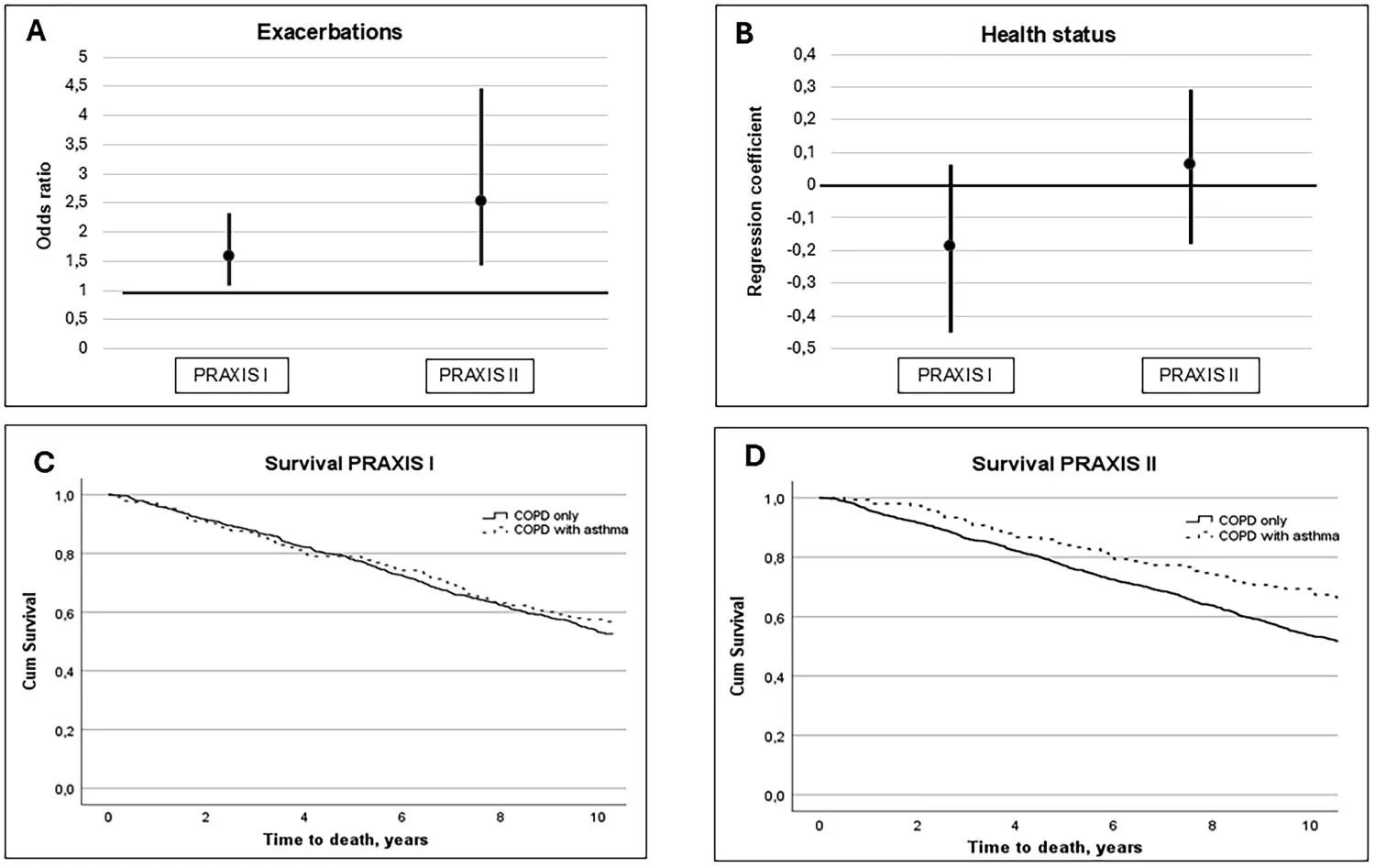
Patient outcomes: exacerbations, health status and survival at follow-up in COPD cohort PRAXIS I and PRAXIS II. Above: comorbid asthma as independent variable. A – logistic regression with dependent variable ‘Exacerbations at follow-up’. B – linear regression with dependent variable ‘Mean CCQ at follow-up’. Adjustments for age, sex, BMI, comorbid heart disease, smoking, exacerbations and CCQ, all at baseline. Below: cumulative 10-year survival in patients with COPD with and without comorbid asthma. C – PRAXIS I, survival 2005–2015. D – PRAXIS II, survival 2014–2024. CCQ: clinical COPD questionnaire.

**Table 2.** Outcomes of health status and exacerbations at follow-up in COPD cohort PRAXIS I and PRAXIS II in 2012 and 2022, respectively, and 10-year mortality in 2015 and 2024.

	Linear regression dependent variable – CCQ at follow-up adjusted data				Logistic regression dependent variable – exacerbations at follow-up adjusted data				Cox regression dependent variable – deceased adjusted data			
	PRAXIS I		PRAXIS II		PRAXIS I		PRAXIS II		PRAXIS I		PRAXIS II	
Patient characteristics at baseline	CCQ Regr coeff (95% CI)	*p* Value	CCQ Regr coeff (95% CI)	*p* Value	Exacerbations odds ratio (95% CI)	*p* Value	Exacerbations odds ratio (95% CI)	*p* Value	Deceased 2015 hazard ratio (95% CI)	*p* Value	Deceased 2024hazard ratio (95% CI)	*p* Value
Comorbid asthma	−0.19 (−0.45 to 0.06)	0.14	0.01 (−0.20to 0.27)	0.76	1.58 (1.07–2.32)	0.02	2.20 (1.38–3.52)	<0.001	0.92 (0.71–1.18)	0.49	0.67 (0.49–0.93)	0.02
Female sex	0.22 (−0.01 to 0.44)	0.06	0.02 (−0.15 to 0.16)	0.95	1.13 (0.79–1.62)	0.50	0.90 (0.63–1.28)	0.55	0.99 (0.80–1.22)	0.90	1.34 (1.13–1.59)	<0.001
Age groups, years												
≤60	Ref		Ref		Ref		Ref		Ref		Ref	–
61–65	0.13 (−0.15 to 0.51)	0.38	0.46 (−0.10 to 0.39)	0.25	1.03 (0.64–1.64)	0.92	0.62 (0.34–1.14)	0.12	2.22 (1.58–3.16)	<0.001	1.76 (1.23–2.52)	<0.001
66–70	0.11 (−0.17 to 0.39)	0.45	0.10 (0.034 to 0.46)	0.02	0.88 (0.55–1.41)	0.58	0.80 (0.49–1.30)	0.36	2.15 (1.54–3.00)	<0.001	2.15 (1.58–2.94)	<0.001
>70	0.46 (0.12–0.80)	0.01	0.10 (0.05 to 0.50)	0.02	0.97 (0.5–1.61)	0.90	0.61 (0.37–1.01)	0.06	2.67 (1.90–3.73)	<0.001	2.92 (2.15–3.97)	<0.001
Comorbid heart disease	0,003 (−0.30 to 0.31)	0.99	0.03 (−0.11 to 0.30)	0.38	0.65 (0.42–1.00)	0.05	0.73 (0.47 –1.14)	0.17	1.76 (1.41–2.19)	<0.001	1.44 (1.19–1.73)	<0.001
CCQ at baseline	0.66 (0.57–0.75)	<0.001	0.68(0.67 to 0.82)	<0.001	1.13 (0.98–1.30)	0.09	1.03 (0.89–1.20)	0.67	1.21 (1.11–1.31)	<0.001	1.35 (1.26–1.45)	<0.001
Exacerbations at baseline	0.31 (0.06–0.56)	0.02	0.05 (−0.41 to 0.34)	0.13	1.89 (1.30–2.76)	<0.001	2.32 (1.58–3.42)	<0.001	1.09 (0.87–1.36)	0.46	1.25 (1.04–1.51)	0.20
Smoking status												
Never smoker	−0.35 (−0.80 to 0.11)	0.14	0.03 (−0.20 to 0.57)	0.35	1. 06 (0.51–2.23)	0.87	1.22(0.50–3.01)	0.66	0.70 (0.41–1.21)	0.20	0.59 (0.34–1.03)	0.06
Ex-smoker	−0.04 (−0.2 to 0.21)	0.76	0.04 (−0.10 to 0.28)	0.35	0.83 (0.56–1.23)	0.35	1.24(0.80–1.93)	0.33	1.06 (0.83–1.35)	0.64	1.12 (0.91–1.37)	0.30
Occasional smoker	0.26 (−0.21 to 0.74)	0.28	0.01 (- 0.27 to 0.39)	0.70	0.56 (0.25–1.25)	0.16	2.71 (1.40–5.27)	0.003	1.38 (0.94–2.04)	0.10	1.02 (0.70–1.48)	0.92
Current daily smoker	Ref		Ref		Ref		Ref		Ref	Ref	Ref	Ref
Body mass index												
<20	0.18 (−0.25 to 0.62)	0.88	0.001 (−0.39 to 0.40)	0.97	1.36 (0.77–2.38)	0.30	0.47 (0.22–1.01)	0.05	0.65 (0.48–0.88)	0.005	2.07 (1.57–2.72)	<0.001
20–24.9	Ref	0.02	Ref	0.33	Ref	0.50	Ref	0.91	Ref	<0.001	Ref	0.002
25–29.9	−0.26 (−0.51 to −0.02)	0.20	0.04 (−0.09 to 0.28)	0.03	1.15 (0.77–1.74)	0.30	0.98(0.65–1.48)	0.32	0.58 (0.58–0.42)	0.049	0.72 (0.58–0.88)	0.006
≥30	−0.15 (−0.45 to 0.15)	–	−0.08 (−0.44 to −0.02)	–	1.29 (0.80–2.09)	–	0.79 (0.50–1.26)	–	0.70 (0.49–1.00)	–	0.73 (0.58–0.92)	–

CCQ: clinical COPD questionnaire; 95% CI: 95% confidence interval.

Comorbid asthma as independent variable.

Linear regression with dependent variable ‘Mean CCQ at follow-up’, logistic regression with dependent variable ‘Exacerbations at follow-up’ and Cox regression with dependent variable ‘Deceased 2015 and deceased 2024’. Adjustments for age, sex, body mass index, comorbid heart disease, smoking, exacerbations and CCQ, all at baseline.

## Discussion

The main findings of this real-world study of patients with a physician’s diagnosis of COPD, mostly from primary care showed that the prevalence of comorbid asthma in a population of patients with COPD was significantly lower in the later PRAXIS II cohort. Furthermore, we found that comorbid asthma was associated with a higher risk of future exacerbations. However, no associations were found between comorbid asthma and worsening of health status or increased risk of mortality.

### Prevalence

We noted a decline in the percentage of patients with COPD and comorbid asthma, from 22% in 2005 to 9% in 2014. The prevalence of COPD with comorbid asthma varies across studies, complicating comparisons due to differences in methods and definitions. A meta-analysis reports that 27% of COPD patients exhibit features of asthma in population-based studies [25]. Our study, however, included patients with a physician’s diagnosis of COPD and asthma, reflecting real-world clinical practice. Other studies using similar diagnostic inclusion criteria as in our study report somewhat different findings: a Majorcan real-world cohort found 18% of patients with COPD had comorbid asthma [14], while two different UK primary care cohorts reported 14.5 and 11%, respectively [10,13].

The considerable difference in prevalence in the two cohorts (22% versus 9%) likely reflects the diagnostic challenges associated with the assessment of obstructive pulmonary diseases where diagnostic confusion can lead to both overdiagnosis and underdiagnosis of asthma and COPD [34,35]. Precision in diagnostics is an ongoing issue which will need continuous attention. Nonetheless, during the past two decades, there has been increased recognition of COPD with repeatedly updated international and national guidelines as well as several national and regional educational activities. We speculate that the focus on diagnostic procedure in guidelines has facilitated the determination of correct diagnoses, potentially explaining the lower prevalence of comorbid asthma in the PRAXIS II cohort.

Accessible spirometry testing in primary care for diagnosing asthma and COPD is important [36]. In Sweden, COPD is mainly managed in primary care where access to spirometry is high. In our cohort, 99% of primary care units had access to spirometry. In a previous study comparing the same cohorts as in this study, we found that the proportion of patients undergoing spirometry increased significantly between 2005 and 2014 [37]. Furthermore, the proportion of correctly interpreted spirometries also increased significantly. This improvement in diagnostic spirometry may partly explain the finding of fewer patients recorded as having COPD and comorbid asthma in the PRAXIS II cohort, due to better diagnostic procedures.

### Exacerbations

In both cohorts, comorbid asthma was associated with an increased risk of exacerbations. This applies for patient characteristics at baseline as well as at follow-up. Several studies have found similar results among patients with COPD who also exhibit features of asthma [16–20,26]. These studies apply the term ACO with varying definitions of the condition. However, our results are also consistent with a study from the U.S.A. with similar inclusion criteria to ours. In this study, patients with a doctor’s diagnosis of both asthma and COPD were more likely to have respiratory-related outpatient or emergency department visits, compared to those with COPD alone [38]. In contrast, in a COPD cohort of university hospital outpatients with a self-reported doctor’s diagnosis of asthma, Caillaud et al. [21] found that patients with both diagnoses did not have more exacerbations.

It could seem logical to assume having both diseases would increase the risk of exacerbations. Furthermore, a history of exacerbations is a major determinant of future exacerbations in patients with COPD [39]. On the other hand, we do not know from our data the underlying cause of exacerbations. One could presume that if the asthma component was optimally treated the risk of exacerbations might decrease. We found that a significantly larger proportion of patients with both COPD and asthma used ICS in both cohorts, ∼79% with comorbid asthma versus 53% with COPD only (Table 1). Nevertheless, if an even larger proportion of patients with both COPD and asthma used ICS regularly it might have affected the exacerbation outcome.

### Health status, CCQ

In our study, health status at baseline did not differ between patients with COPD only and COPD with comorbid asthma. A similar finding is presented in a Scandinavian real-world out-patient population and in an Australian primary care cohort where patients with a diagnosis of both asthma and COPD did not report worse health status than those with COPD only [40,41].

We found comorbid asthma was not associated with a worsening of health status over a follow-up period of seven (PRAXIS I) and eight years (PRAXIS II). A similar finding is reported by Fu et al. [42] where patients with both COPD and asthma did not experience a significant decline of health status after four years follow-up. Opposed to this, in a hospital-based cohort, Kauppi et al. [43] report worse health status in individuals with both diagnoses.

Previous studies have shown deterioration in health status over time in patients with COPD only [42,44]. A possible explanation for our results is that COPD itself gradually causes a decline in health status, and the presence of asthma does not add to this deterioration. Additionally, patients with COPD and comorbid asthma use more ICS than those with COPD only (Table 1). This suggests that the asthma component of their condition could be well managed, which may help reduce its impact on their health status. This again emphasizes the burden of COPD itself on health status [45].

### Mortality

We found that comorbid asthma did not alter the risk of mortality in any way in the PRAXIS I cohort, whereas in PRAXIS II, it was associated with a reduced mortality risk. Previously reported data on mortality are inconsistent with some studies showing unchanged [11,20,21], increased [22] or diminished [23,26] mortality rates in patients with COPD and comorbid asthma compared to those with COPD alone. Baarnes et al. [22] performed a large cohort study where patients were included upon hospital admission, which suggests a population with more severe disease than ours. Another hospital based, but smaller, Finnish register-study with a 10-year follow-up report a significantly higher survival of patients with a diagnosis of both COPD and asthma compared to COPD only [23]. In a Swedish observational study of COPD patients in primary care, comorbid asthma was also associated with a decreased the risk of mortality [26]. As before mentioned, variations in methods and definitions likely contribute to these discrepancies in outcomes.

We speculate that the different outcomes between the two PRAXIS cohorts may reflect more appropriate asthma diagnostics and management in the later cohort. It is possible that patients in the PRAXIS I cohort who were diagnosed with comorbid asthma may, in fact, have had COPD alone. If so, the ‘COPD + asthma’ group would have a poorer prognosis due to their COPD diagnosis. Differences in the use of ICS among patients with comorbid asthma could indicate better clinical assessment and a reduced risk of mortality. However, no differences in ICS use were observed at baseline between the cohorts (Table 1). An *ad hoc* analysis of patients with COPD and comorbid asthma at follow-up (2012 and 2022) confirms this finding (data not shown). Additionally, a sensitivity regression analysis adjusting for ICS use did not alter the results.

### Strengths and limitations

Major strengths of this cohort study are the large sample sizes and the real-world data from medical reviews and questionnaires with a relatively high frequency of responders. This contributes to a high generalizability and external validity in areas with similar health care systems. Other strengths are the follow-up times of seven and eight years, respectively, concerning self-reported health status and exacerbations, and the 10-year follow-up of mortality outcome.

Limitations include missing data, especially on spirometry in the 2005 cohort. This could lead to selection bias in categorization of patients in groups according to FEV1% pred. On the other hand, spirometry data were not needed to assess the outcomes in this study and lack of spirometry data did not affect the sample size. Another limitation is the uncertainty regarding the reliability of the diagnosis, particularly the asthma diagnosis, as asthma can be difficult to diagnose due to its varying clinical manifestations. Self-reported data could always be subject to recall bias.

## Conclusion

The prevalence of comorbid asthma has changed from 22 to 9% in two different COPD cohorts, likely reflecting improved diagnostic accuracy.

Nonetheless, the presence of asthma in COPD remains clinically relevant due to its association with increased exacerbation risk, underscoring the importance of accurate diagnosis and management.

## Data Availability

Data cannot be made freely available as they are subject to secrecy in accordance with the Swedish Public Access to Information and Secrecy Act, but can be made available to researchers upon request, after approval from the Swedish Ethical Review Authority has been obtained.
